# Effect of antipsychotic consumption during pregnancy on risk of gestational diabetes development: a systematic review and meta-analysis

**DOI:** 10.3389/fpsyt.2025.1710101

**Published:** 2026-02-10

**Authors:** Jingna Liu, Yanyan Zhao

**Affiliations:** Neurology Department, Guangzhou Red Cross Hospital, Guangzhou, China

**Keywords:** antipsychotics, pregnancy, gestational diabetes mellitus, meta-analysis, psychotropic medications

## Abstract

**Background:**

The use of antipsychotic medications, including first-generation antipsychotics (FGAs) and second-generation antipsychotics (SGAs), during pregnancy has risen substantially. However, concerns remain regarding their potential metabolic effects, especially the risk of gestational diabetes mellitus (GDM).

**Methods:**

We conducted a systematic review and meta-analysis of observational studies published up to 2025 that examined the association between maternal exposure to antipsychotics or antidepressants and the risk of GDM. Data were extracted independently by two reviewers, quality was assessed using the Newcastle–Ottawa Scale, and pooled relative risks (RRs) were calculated using a random-effects model.

**Results:**

We selected seventeen eligible studies, including large registry-based cohorts and prospective investigations across multiple countries. Pooled analysis demonstrated that maternal exposure to SGAs was significantly associated with an increased risk of GDM (RR = 1.59; 95% CI: 1.24–1.94), whereas FGAs showed no significant association (RR = 1.31; 95% CI: 0.29–2.32). The risk appeared greatest among women with continuous or multi-trimester exposure, particularly when exposure extended from first trimester into the third trimester. Funnel plot and Egger test showed no publication bias.

**Conclusions:**

The higher risk of GDM seen with antipsychotic use was mainly linked to SGAs, while FGAs did not show the same effect. These findings emphasize the need to tailor treatment plans, closely monitor blood sugar levels, and involve a team of healthcare professionals when caring for pregnant women who require psychotropic medications.

## Introduction

Gestational diabetes mellitus (GDM) is one of the most common metabolic problems that can happen during pregnancy. It can happen in up to 20% of pregnancies around the world, depending on the population and the criteria used to diagnose it (1). Globally, among women of childbearing age, the age-standardized prevalence of diabetes nearly doubled between 1990 and 2021, with the highest burden in low- and low-middle sociodemographic index regions, according to recent global burden of diabetes analysis. This emphasizes the need for targeted prevention policies and highlights a disproportionate rise in diabetes-related morbidity that disproportionately impacts maternal health in lower-resource settings (2). In parallel, the use of psychotropic medications during pregnancy, especially antipsychotics, has increased significantly over the past decade (3). Simultaneously, the use of antipsychotics, particularly second-generation antipsychotics (SGAs), has also risen due to expanded indications, including bipolar disorder and schizoaffective disorders (4). These medications are essential for managing psychiatric conditions and preventing relapses during pregnancy; however, they are known to carry metabolic side effects such as weight gain, insulin resistance, and dyslipidemia, raising concerns about their potential contribution to GDM.

Several observational studies have explored the association between antipsychotic use during pregnancy and the risk of GDM. Evidence suggests that SGAs with high metabolic risks, such as olanzapine, clozapine, and quetiapine, are significantly associated with increased GDM incidence (5). For instance, a large Swedish registry-based study demonstrated that continued use of metabolically high-risk SGAs during pregnancy was linked to nearly a twofold increased risk of GDM compared to discontinuation or use of lower-risk agents (4). However, results from other studies are less consistent. When controlling for demographic and psychiatric comorbidities and socioeconomic factors, some analyses found no statistically significant increase in GDM risk (6). This discrepancy raises the possibility of confounding by indication, as women prescribed antipsychotics may already possess a higher baseline risk for metabolic disorders.

Given these mixed findings and the high prevalence of antipsychotic medications use in pregnant populations, there is a pressing need to clarify the extent to which these medications contribute to GDM risk. In real-world clinical practice, however, many patients are exposed to both types of FGA and SGA drugs, and a comprehensive understanding of their impact on GDM is essential. Therefore, the present study aims to conduct a meta-analysis of available literature from the past decade to evaluate the risk of GDM associated with maternal use of antipsychotics during pregnancy. This work seeks to differentiate the risks posed by drug class and specific agents and provide clinically valuable evidence that can inform risk-benefit decisions and tailored care for pregnant women who need antipsychotic therapy.

## Methods

### Study design

This systematic review and meta-analysis was conducted in accordance with the Preferred Reporting Items for Systematic Reviews and Meta-Analyses (PRISMA) guidelines (7) The methodology included a comprehensive literature search, predefined eligibility criteria following the PICOS framework, a two-stage study selection process, standardized data extraction, independent quality assessment, and appropriate statistical synthesis.

### Search strategy

A systematic search of PubMed, Scopus, and Web of Science was performed to identify studies evaluating the association between maternal use of antipsychotic medications during pregnancy and the risk of GDM. The search covered studies published through June 2025. A combination of Medical Subject Headings (MeSH) and free-text keywords (e.g., “pregnancy,” “antipsychotic,” “olanzapine,” “quetiapine,” “clozapine,” “gestational diabetes”) was used with Boolean operators. Filters restricted results to human studies and articles in English or Chinese. Duplicate records were removed prior to screening.

### Eligibility criteria

Eligibility criteria were defined *a priori* using the PICOS framework:

- Population: Pregnant women of any age or parity.
- Exposure: Use of antipsychotic medications during pregnancy without combination with other agents, including both FGA and SGA agents, irrespective of dose, formulation, or treatment duration. Only studies that had followed the patients at least up to end of pregnancy were considered eligible.
- Comparator: Pregnant women not exposed to antipsychotic medications. Studies comparing different antipsychotic drugs were eligible if they included a non-exposed reference group.
- Outcome: GDM as defined by study-specific diagnostic criteria, including oral glucose tolerance testing, ICD-9/10 codes, or validated medical record documentation.
- Study Design: Only cohort studies or case–control studies.

We included peer-reviewed studies that reported quantitative effect estimates (odds ratio, risk ratio, or hazard ratio with 95% confidence intervals) or provided sufficient data for their calculation. Exclusion criteria were animal studies, *in vitro* studies, case series, reviews, editorials, conference abstracts, non-English/non-Chinese publications, and articles without original data.

### Study selection

**Figure 1:**
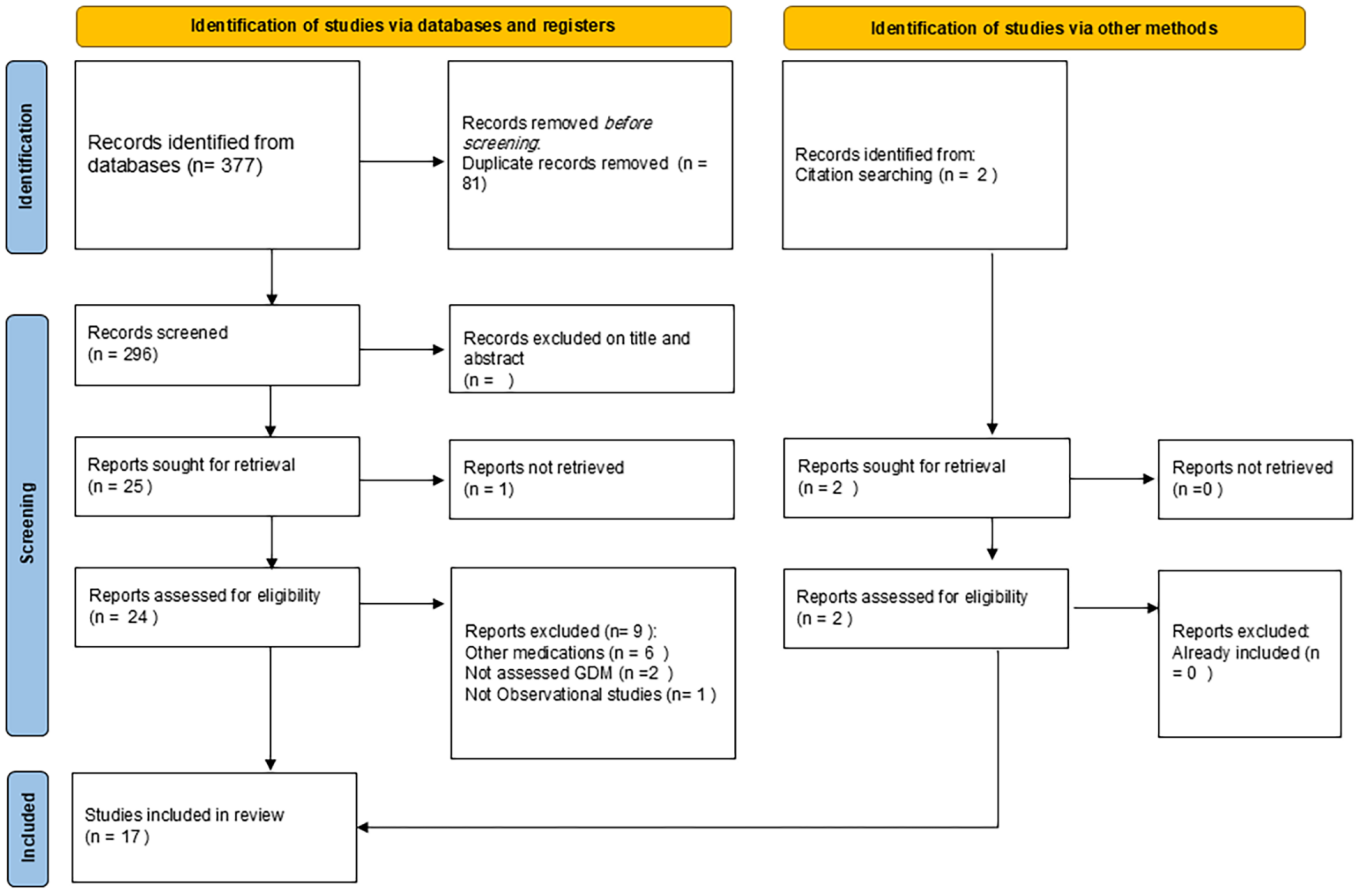
PRISMA diagram.

References were imported into reference management software and deduplicated. Two reviewers independently screened titles and abstracts, followed by a full-text review of potentially eligible studies. Discrepancies were resolved through discussion and reexamination between two authors. Reasons for exclusion were documented, and the selection process was summarized using a PRISMA flow diagram (**Figure 1**).

### Data extraction

Data extraction was carried out using a standardized form by two independent reviewers, who collected information on the first author, year of publication, and journal, along with details on the study design, country, data source, recruitment period, and sample size. They also recorded population characteristics such as maternal age and the inclusion or exclusion criteria applied. Information on exposures included the specific drug or drugs studied, their classification as first- or second-generation antipsychotics, the administered dose, route, and timing by trimester, as well as the method of ascertainment. Outcomes were defined as gestational diabetes mellitus, with diagnostic criteria and ascertainment methods clearly specified. Finally, effect measures were extracted in the form of adjusted odds ratios or relative risks with 95% confidence intervals, prioritizing the most fully adjusted models. Corresponding authors were contacted when additional information was required.

### Quality assessment

Risk of bias was assessed independently by two reviewers using the Newcastle–Ottawa Scale (NOS) for observational studies. This tool evaluates selection, comparability, and outcome domains, with scores up to 9 indicating high quality. NOS scores were incorporated into interpretation and subgroup analyses but were not used as exclusion criteria.

### Statistical analysis

Pooled effect estimates were calculated using a random-effects meta-analysis. Given the relatively low incidence of GDM, odds ratios were treated as approximate relative risks. Between-study heterogeneity was assessed with Cochran’s Q and quantified with the I² statistic (25%, 50%, and 75% corresponding to low, moderate, and high heterogeneity). Sensitivity analyses (using leave one out calculation) were conducted to find the heterogeneous studies. Additional and subgroup analyses included comparisons by antipsychotic class (FGA vs. SGA) and timing of exposure (first, second, third trimester, or entire pregnancy). Publication bias was assessed visually using funnel plots. All statistical analyses were carried out using STATA (version 17) and Revman (version 5).

## Results

### Study characteristics

**Figure 2:**
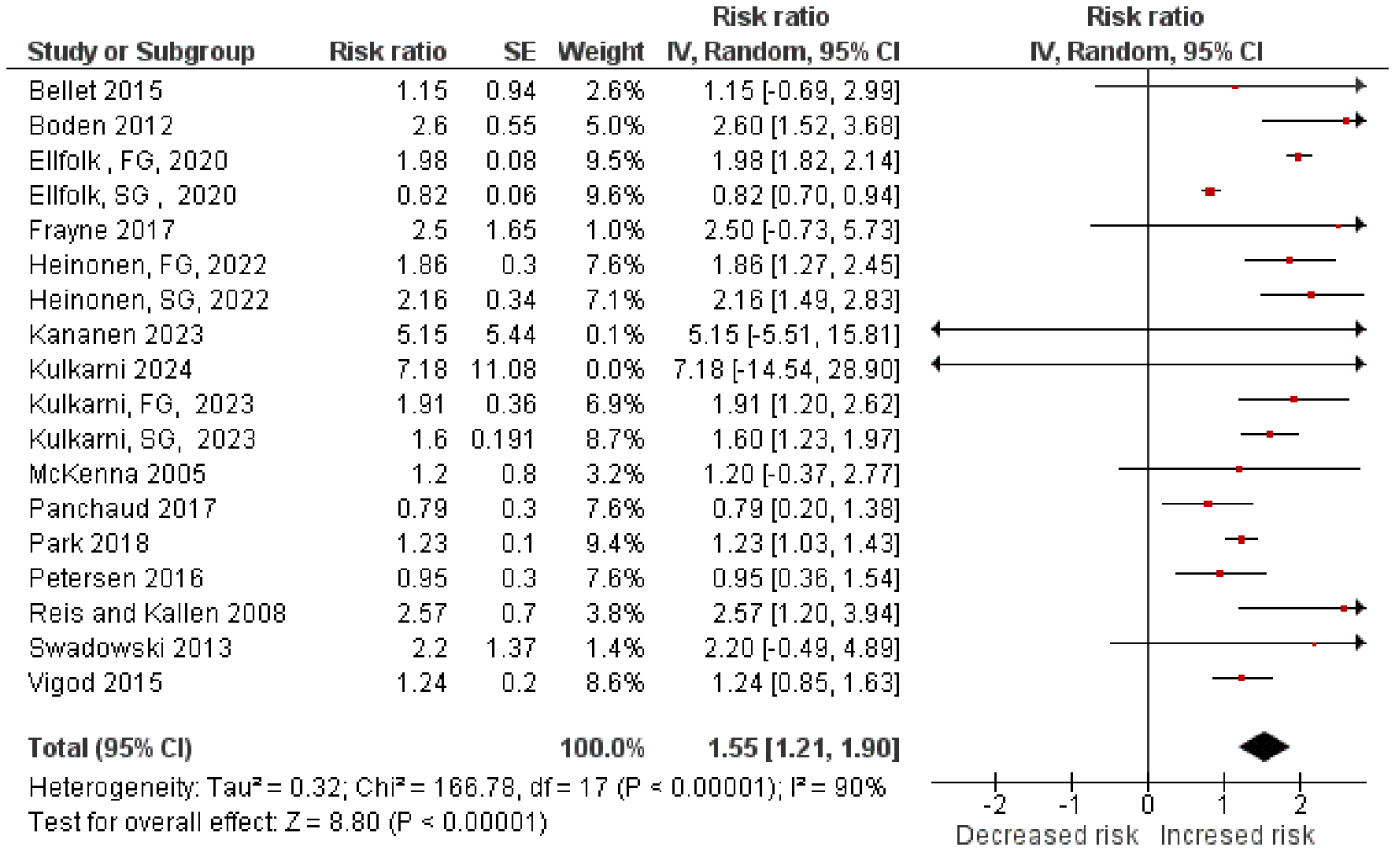
Relative risks (RR) for gestational diabetes mellitus (GDM) associated with antipsychotic exposure (encompassing both first- and second-generation agents) were pooled. For studies that provided discrete data, summary estimates were also generated separately for FGAs and SGAs. In the accompanying forest plot, the suffixes "FG" and "SG" appended to study identifiers distinguish these subgroup-specific pooled relative risks.

Our search yielded 377 articles. After removal of duplicates, title/abstract, and full-text screening, seventeen studies were included in this review (**Figure 2**). The included studies were conducted across multiple countries, including Canada, Israel, the United Kingdom, Sweden, the United States, France, New Zealand, Finland, and Australia. The majority employed retrospective cohort designs, with several prospective cohorts and one case-control study. The primary exposures were FGAs and SGAs, including haloperidol, chlorpromazine, fluphenazine, thioridazine, quetiapine, olanzapine, risperidone, clozapine, and aripiprazole. Drug exposure was most assessed during the entire (all trimesters of pregnancy, although several studies captured second- and third-trimester exposure. GDM was primarily ascertained using ICD-9 or ICD-10 diagnostic codes, chart reviews, and health record linkage. We summarized the design and findings of each study. Further information involving the study locations, designs, drug exposures, timing of medication during pregnancy, and diagnostic criteria for GDM is summarized in **Table 1**. Most studies relied on registry or medical record data, though definitions of exposure windows varied.

**Table 1:** Characteristics of included studies.

Study	Location	Study design	Medications	Trimester of drug used	Exposure window	GDM determined by
McKenna et al., 2005 (8)	Canada, Israel, UK	Prospective, cohort	Olanzapine Risperidone Quetiapine Clozapine	First trimester	Within 3 months of pregnancy or during pregnancy, no detailed description of the pregnancy definition	ICD criteria, Chart reviews, delivery, or health records
Reis and Kallen (2008) (9)	Sweden	Retrospective, cohort	(FGAs): Haloperidol Chlorpromazine Fluphenazine Thioridazine (SGAs): Quetiapine Olanzapine Risperidone Aripiprazole Paliperidone	Early pregnancy (usually before the end of the first trimester), no detailed description for pregnancy definition	31.2 weeks, with a range of 4 to 42 weeks.	ICD criteria
Wichman, 2009 (10)	United States	Retrospective	Quetiapine Risperidone Aripiprazole Ziprasidone	Conception Discontinuation after a positive pregnancy test 1st trimester (0–13 weeks) 2nd trimester (14–26 weeks) 3rd trimester (27 weeks to delivery)r	Most exposures occurred at conception (76.5%) and in 84.6% of cases the medication was continued after the pregnancy was recognized	ICD-10
Boden et al. (2012)	Sweden	Retrospective, cohort	Group 1 (high obesogenic and diabetogenic potential Olanzapine Clozapine **Group 2 (other antipsychotics):** Quetiapine fumarate Risperidone Flupentixol Haloperidol Aripiprazole Perphenazine Zuclopenthixol Ziprasidone hydrochloride Chlorprothixene Fluphenazine Pimozide	From last menstrual period to parturition	Women who exposed to antipsychotics in pregnancy (From last menstrual period to parturition)	ICD 10
Sadowski etal. (2013) (11)	Canada	Prospective, cohort	(SGAs): Quetiapine (69.9%) Olanzapine (16.5%) Risperidone (10.5%) Aripiprazole, Paliperidone, and Quetiapine + Olanzapine (combined 3.1%).	A minimum of 4 weeks during pregnancy, no detailed description for pregnancy definition	Women who confirmed the use of SGAs for a minimum of 4 weeks of pregnancy, no detailed descriptions for whether co-medication with FGAs	ICD-10
Bellet et al. (2015) (12)	France	Prospective, cohort	Aripiprazole	During embryogenesis	During embryogenesis (gestational weeks, i.e. weeks after the last menstrual period)	ICD criteria
Vigod et al. (2015) (13)	Canada	Retrospective, cohort	Dixyrazine, Prochlorperazine, Fluphenazine, Perphenazine. Thioridazine. Haloperidol, Melperone. Flupentixol, Zuclopenthixol, Pimozide. Benzodiazepines & Oxazepines Clozapine, Olanzapine, Quetiapine. Risperidone, Lithium	First or second trimester	Between the conception date and the delivery date, at least one prescription within the first or second trimester	ICD-10
Petersen etal. (2016) (14)	UK	Retrospective, cohort	Fluoxetine Sertraline Citalopram Paroxetine Escitalopram Venlafaxine Duloxetine Amitriptyline Nortriptyline Mirtazapine Trazodone	First Trimester	Between 31 and 105 days (inclusive) after the start of pregnancy (the first day of last menstrual period or 280 days before delivery if no records suggested a different duration of pregnancy)	ICD-10
Friedman et al, 2016 (15)	New Zealand	Retrospective review	Quetiapine: 21 women (47%) Olanzapine: 19 women (42%) Risperidone: 7 women (16%) Aripiprazole: 6 women (13%) Clozapine: 1 woman (2%)	First Trimester	Women were referred and enrolled from the second trimester onwards, though first-trimester medication exposure was recorded from prescription histories	ICD-10
Panchaud etal. (2017) (16)	USA	Prospective, cohort	Olanzapine Clozapine Quetiapine Risperidone	The first trimester, no detailed description for pregnancy definition	No detailed description for pregnancy definition	ICD-10
Frayne etal. (2017) (17)	Australia	Retrospective, cohort	not report	The third trimester, no detailed description for pregnancy definition	No detailed description for pregnancy definition	ICD-10
Park, 2018 (18)	usa	Retrospective, cohort	Quetiapine: 58.8% Aripiprazole: 25% Risperidone: 23.7% Olanzapine: 18.5% Ziprasidone: 8.7%	First half of pregnancy, the first 140 days, approximately 20 weeks	First 140 days of pregnancy (pregnancy defined as last menstrual period to the date of delivery)	icd-10
Ellfolk, 2020 (19)	Finland	cohort study, Retrospective	Quetiapine Olanzapine Risperidone Aripiprazole Clozapine Ziprasidone Sertindole Asenapine	First trimester, second trimester, third trimester	From one month before the start of pregnancy until the end of pregnancy	ICD criteria
Heinonen, 2022 (20)	Sweden	Cohort Study,retrospective	levomepromazine Haloperidol Flupentixol Quetiapine Olanzapine Clozapine Aripiprazole Risperidone Ziprasidone Paliperidone Sertindole	First trimester , second trimester , third trimester	Taking the drug at any time from one month before pregnancy until the end of pregnancy ,Receiving medication from one month before to a maximum of 90 days before the last day of pregnancy ,Receiving medication in the last 90 days of pregnancy, with or without prior use	ICD criteria
Kananen, 2023 (21)	Finland	cohort	Quetiapine Olanzapine Perphenazine Prochlorperazine Clozapine Risperidone Chlorprothixene Haloperidol Chlorpromazine Levomepromazine Aripiprazole Thioridazine Melperone Promazine	First trimester, second trimester, third trimester	All women in the groups used (FGA and SGA) had taken antipsychotic medications at least during the first trimester of pregnancy (first 13 weeks)	ICD criteria, Chart reviews, delivery, or health records
J. Kulkarni, 2023 (22)	Australia	Prospective cohort	Olanzapine Quetiapine Clozapine Paliperidone Aripiprazole Ziprasidone Consta Risperidone Asenapine	First trimester	Within first 3 months during pregnancy, no detailed description for pregnancy definition	ADIPS
Kulkarni, 2024 (23)	Australia	Case-control study	Clozapine , quetiapine	First trimester	Within the first 3 months of, no detailed description of pregnancy definition	Not reported

ICD, International Classification of Diseases; FGA, First-generation antipsychotics; SGA, Second-generation antipsychotics; GDM, Gestational diabetes mellitus.

McKenna et al. (2005): This multinational prospective cohort conducted across Canada, Israel, and the UK, focusing on the use of SGAs such as olanzapine, risperidone, quetiapine, and clozapine during the first trimester of pregnancy. Exposure was determined through maternal reports and medical documentation, while GDM was identified using ICD criteria, chart reviews, and delivery or health records (8).

Reis and Källén (2008): A retrospective cohort study carried out in Sweden examining the effects of both FGAs, including haloperidol, chlorpromazine, fluphenazine, and thioridazine, and SGAs, such as quetiapine, olanzapine, risperidone, aripiprazole, and paliperidone. Exposure was recorded during early pregnancy, usually before the end of the first trimester, although no detailed description of pregnancy definition was provided. GDM outcomes were determined using ICD criteria (9).

Wichman (2009): A retrospective study conducted in the United States assessing exposure to SGAs such as quetiapine, risperidone, aripiprazole, and ziprasidone. Medication use was considered at conception, discontinuation after a positive pregnancy test, or across the first, second, and third trimesters. Outcomes were identified using ICD-10 diagnostic codes (10).

Boden et al. (2012): A retrospective cohort study performed in Sweden that grouped antipsychotics according to their obesogenic and diabetogenic potential. Effects of both SGAs such as olanzapine, clozapine, quetiapine, risperidone, aripiprazole, ziprasidone and FGAs such as Flupentixol, Haloperidol, Perphenazine, Zuclopenthixol, Chlorprothixene, Fluphenazine, Pimozide were examined. Exposure was measured from the last menstrual period until delivery, and outcomes were determined using ICD-10 criteria (4).

Sadowski et al. (2013): A prospective cohort conducted in Canada evaluating SGAs such as quetiapine, olanzapine, risperidone, aripiprazole, paliperidone during pregnancy. Exposure was defined as at least four weeks during pregnancy, although no detailed description of pregnancy definition was provided. Outcomes were identified using ICD-10 codes (11).

Bellet et al. (2015): A prospective cohort carried out in France focusing on exposure to SGAs such as aripiprazole during embryogenesis, defined as the gestational weeks following the last menstrual period. GDM outcomes were determined using ICD criteria (12).

Vigod et al. (2015): A large retrospective cohort study was conducted in Canada examining exposure to a wide range of antipsychotics, including FGAs such as dixyrazine, prochlorperazine, fluphenazine, perphenazine, thioridazine, haloperidol, melperone, flupentixol, zuclopenthixol, pimozide and SGAs such as clozapine, olanzapine, quetiapine, risperidone, lithium, and benzodiazepines. Women were considered exposed if they received at least one prescription within the first or second trimester, from conception to delivery. GDM was identified using ICD-10 codes (13).

Petersen et al. (2016): A UK retrospective cohort focusing antipsychotic exposure and GDM risk Exposure was defined as use of antipsychotics between 31 and 105 days after the beginning of pregnancy, calculated from the first day of the last menstrual period or, if unavailable, 280 days prior to delivery, unless other records indicated a different gestational duration. Outcomes were identified using ICD-10 diagnostic codes (14).

Friedman et al. (2016): A retrospective review carried out in New Zealand of women referred from the second trimester onward, although first-trimester exposure was also recorded from prescription histories. The effect of SGAs such as quetiapine, olanzapine, risperidone, aripiprazole, clozapine were examined. Outcomes were identified using ICD-10 diagnostic codes (15).

Panchaud et al. (2017): A U.S. prospective cohort with first-trimester SGAs such as olanzapine, clozapine, quetiapine, risperidone. No detailed pregnancy definition was provided. Outcomes were determined using ICD-10 criteria (16).

Frayne et al. (2017): A retrospective cohort study conducted in Australia evaluating antipsychotic use during the third trimester of pregnancy, although the specific medications were not reported. Outcomes were identified using ICD-10 diagnostic codes (17).

Park (2018): A retrospective cohort conducted in the United States examining antipsychotic use during the first half of pregnancy, defined as approximately the first 140 days or 20 weeks. The effects of SGAs such as quetiapine, aripiprazole, risperidone, olanzapine, and ziprasidone were examined. GDM outcomes were determined using ICD-10 codes (18).

Ellfolk (2020): A Finnish registry-based cohort including SGAs and FGAs such as quetiapine, olanzapine, risperidone, aripiprazole, clozapine, ziprasidone, sertindole, asenapine, with exposure classified across all trimesters. The use of national registry data enhanced reliability. Outcomes were determined using ICD criteria (19).

Heinonen (2022): A large retrospective cohort study in Finland focusing on women exposed to a broad spectrum of FGAs such as levomepromazine, haloperidol, flupentixol and SGAs such as quetiapine, olanzapine, clozapine, aripiprazole, risperidone, ziprasidone, paliperidone, sertindole. Exposure was documented across all trimesters, and outcomes were identified using ICD criteria, chart reviews, and delivery or health records (20).

Kananen (2023): A cohort study conducted in Finland focusing on women exposed to a broad spectrum of FGAs such as perphenazine, prochlorperazine, chlorprothixene, haloperidol, chlorpromazine, levomepromazine, thioridazine, melperone, promazine and SGAs such as quetiapine, olanzapine, clozapine, risperidone, aripiprazole. Exposure was documented across all trimesters, and outcomes were identified using ICD criteria, chart reviews, and delivery or health records (21).

Kulkarni (2023): An Australian prospective cohort examining SGAs, including olanzapine, quetiapine, clozapine, paliperidone, aripiprazole, ziprasidone, risperidone, asenapine, risperidone consta during the first trimester. GDM was determined according to ADIPS criteria (22).

Kulkarni (2024): A recent case-control study focusing on SGAs such as clozapine and quetiapine exposure during the first trimester. Outcomes related to GDM were not specifically reported (23).

### Main meta-analytic findings

The pooled evidence from included studies demonstrated variable associations between antipsychotic exposure during pregnancy and the subsequent risk of GDM. In each study, RRs varied widely, reflecting differences in the study design as well as types of antipsychotics (FGA vs SGA). Bellet (2015) (12) and McKenna (2005) (8) reported modest or non-significant associations. For instance, Bellet (2015) reported a RR of 1.15 and McKenna (2005) reported a RR of 1.20. Panchaud et al. (16) in 2017 reported RR: 0.79, and Petersen et al. (14) reported RR 0.95; no clear evidence of elevated risks has been demonstrated. However, other studies found a stronger positive correlation. Boden (2012) (4) reported a significant two- to three-fold increase (RR = 2.6), while Reis and Källén (2008) (9) also found elevated risk (RR = 2.57). More recent data by Park (24) (2018; RR = 1.23) suggested a modest but statistically significant increase. The results of Ellfolk (2020) (19) and Heinonen (2022) (20) were in agreement. Regarding FGAs, Ellfolk (2020) (19) demonstrated a nearly two-fold risk (RR = 1.98), and Heinonen (2022) (20) confirmed this finding (RR = 1.86). Additionally, Kulkarni (2023) (22) supports this finding (RR = 1.91). These findings indicate that FGA use is associated with an increased risk of GDM. SGAs, on the other hand, presented a more complex picture. Ellfolk (2020) (19) found a protective association (RR = 0.82), suggesting a reduced risk, whereas Heinonen (20) (2022 RR = 2.16) and Kulkarni (22) (2023 RR = 1.60) both indicated increased risk. There may be differences between SGA subtypes or study populations, which highlights the need for careful interpretation. Studies with wide CIs, such as Frayne (17) (2017; RR = 2.50), Sadowski (11) (2013; RR = 2.20), Kananen (21) (2023; RR = 1.91), and Kulkarni (23) (2024; RR = 7.18), were inconclusive due to high imprecision, suggesting limited reliability of their estimates.

In our meta-analysis, the pooled RR for risk of GDM in antipsychotic users was 1.55 (95% CI: 1.21–1.81), suggesting a significant risk of GDM development in mothers who use antipsychotics during their pregnancy.

### Sensitivity analysis, subgroup analysis, and meta-regression

**Figure 3:**
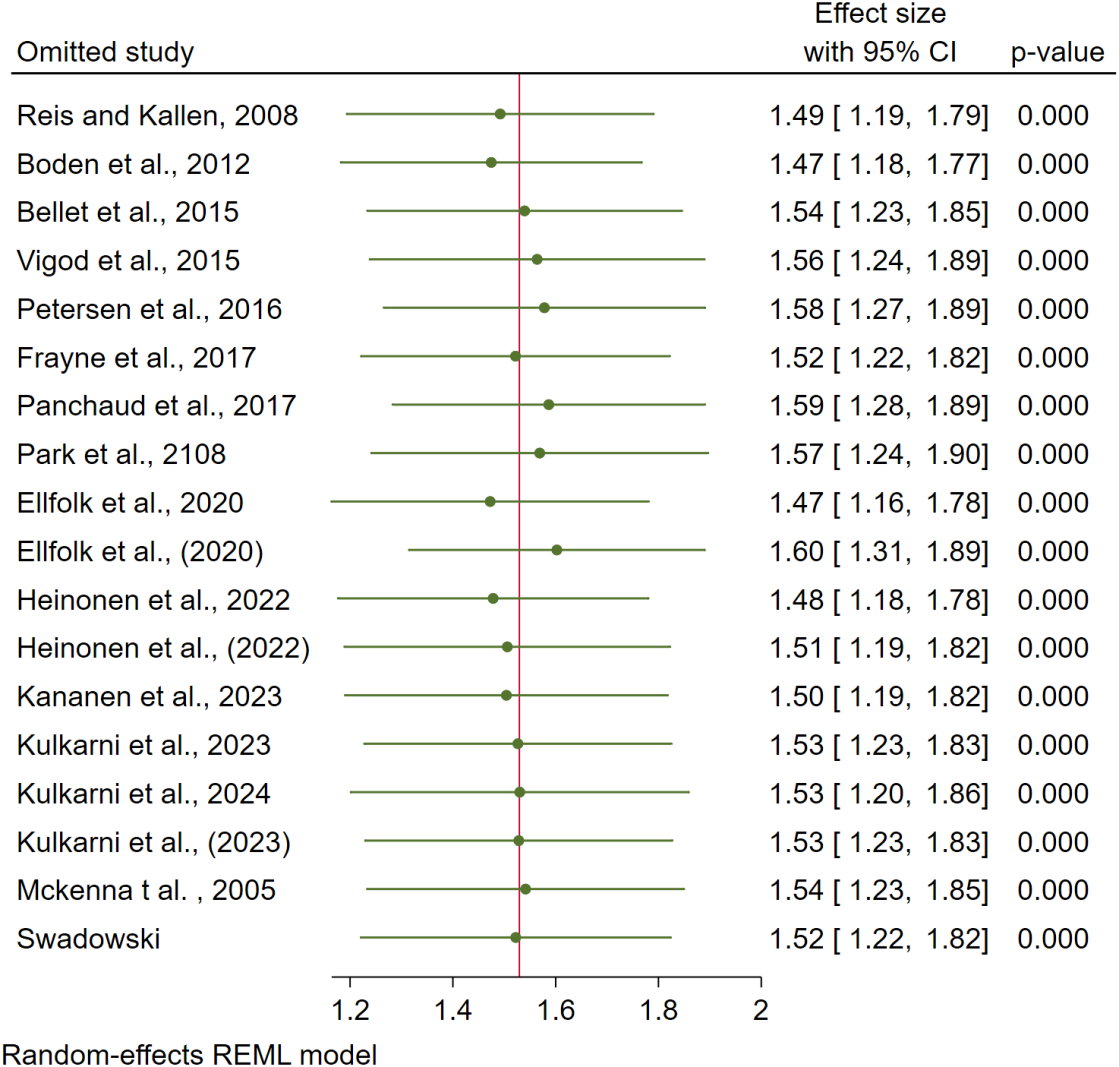
Sensitivity analysis using leave-one-out methode.

Using leave-one-out calculation, a sensitivity analysis was performed to find the effect of each study on the pooled effect size (**Figure 3**). Subgroup meta-analysis was carried out based on the generation of antipsychotics and the trimesters of exposure. Pooled analysis showed that exposure to SGAs was associated with a statistically significant increase in GDM risk (RR = 1.59, 95% CI: 1.24–1.94), whereas FGAs demonstrated a non-significant association (RR = 1.31, 95% CI: 0.29–2.32). The results for each subgroup and associated I^2^ are indicated in **Table 2**. Besides sensitivity analysis and subgroup analysis, the heterogeneity remained high in some groups. To further investigate the potential sources of heterogeneity observed in some subgroups, we conducted univariable meta-regression analyses using study-level characteristics as covariates. The moderators examined included study design (prospective cohort, retrospective cohort, or case-control), geographical location (Asia, Europe, or America), sample size, and generation of antipsychotic drugs. All models were fitted using the restricted maximum likelihood (REML) method.Meta-regression by study design yielded a residual τ² = 0.2403 and I² = 86.17%, with *R²* = 0.00%. The association between study design and the pooled effect size was not statistically significant (Wald χ² = 0.01, *p* = 0.9062), indicating that study design did not explain the between-study variability. Meta-regression including sample size (number of observations = 18) produced residual τ² = 0.2462 and I² = 85.01%, with *R²* = 0.00%. The covariate was not a significant predictor of effect estimates (Wald χ² = 0.10, *p* = 0.7565), suggesting that differences in sample size did not contribute meaningfully to heterogeneity. When continent was entered as a moderator, residual τ² = 0.193 and I² = 81.70%, with *R²* = 11.18%. The relationship between geographic region and effect size was not significant (Wald χ² = 2.54, *p* = 0.1109). Although the explained heterogeneity was modest, this model suggested a slight trend toward lower heterogeneity compared with the overall analysis. Overall, none of the examined study-level covariates significantly explained the between-study heterogeneity. These findings suggest that the observed variability may arise from unmeasured methodological or population-specific factors. Although exploratory meta-regression did not identify significant moderators, it reinforces the need for future primary studies with harmonized designs and standardized exposure measures to clarify sources of heterogeneity.

**Table 2:** Results of subgroup meta-analyses of antipsychotic exposure and GDM risk based on the timing of exposure and generation of drugs.

Subgroup title	Number of included studies in subgroup analysis	Effect size (risk ratio)	Confidence intervals	Heterogeneity (I^2^)
FGAs	3	1.31	0.29-2.32	83%
SGAs	10	1.59	1.24-1.94	75%
Exposure in the first trimester	5	1.25	0.63-1.87	44%
Exposure in the third trimester	1	2.5	0.73-5.73	0%
Exposure in the first and second trimesters	1	1.24	0.84-1.64	0%
Exposure in all trimesters	6	1.52	1.16-2.24	85%

FGA, First-generation antipsychotics; SGA, Second-generation antipsychotics.

### Publication bias

**Figure 4:**
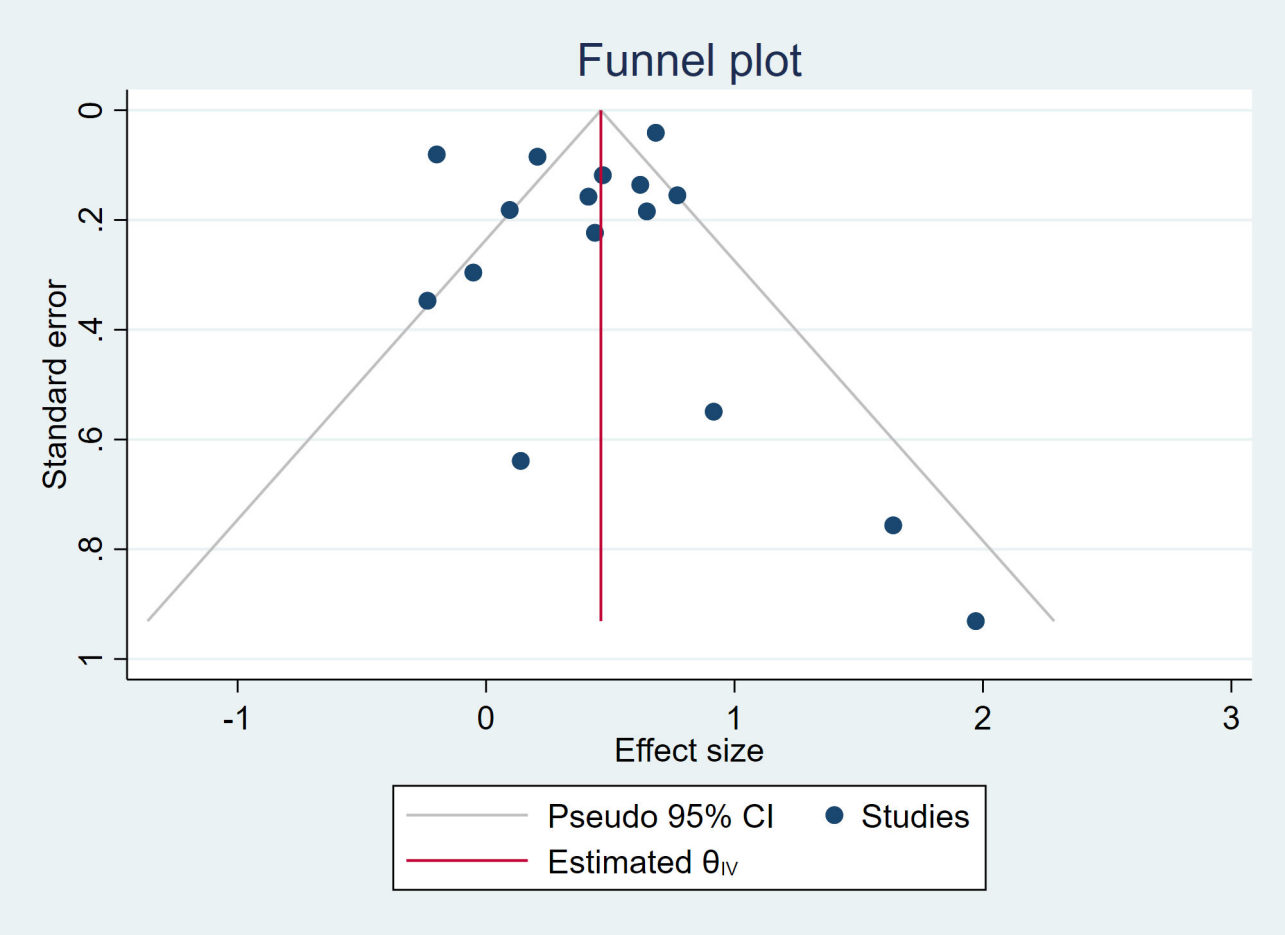
Funnel plot of included studies.

**Figure 4** displays a funnel plot assessing small-study effects. The plot was largely symmetrical, indicating a low likelihood of significant publication bias. However, minor asymmetry at the plot extremes cannot be fully excluded due to the limited number of studies included. Most of the included studies reported very low standard errors (SEs), with estimates clustering at the top of the funnel plot. This pattern is likely explained by the use of large registry-based datasets, which provide substantial sample sizes and consequently more precise effect estimates. The Egger test showed that there is no publication bias or small-study effects (beta1 = 0.73, SE of beta1 = 0.453). The Egger’s test and funnel plot should be interpreted cautiously, due to limited number of included studies.

### Risk of bias assessment

**Table 3** summarizes the risk of bias for each included study, evaluated using NOS. Many studies were rated as having a low risk of bias, particularly with respect to selection and outcome ascertainment. Two recent studies (Kulkarni et al, 2023 (22) and Kulkarni et al, 2024 (23)) were rated as having a moderate risk of bias owing to limited comparability and small sample sizes. Overall, the evidence base is strengthened by the inclusion of several large, population-based cohorts, though variability in exposure definitions and potential residual confounding remain important considerations.

**Table 3:** Newcastle–Ottawa Scale quality assessment.

Study	Selection	Comparability	Outcome	Total score	Risk assessment
McKenna	4	1	3	8	Low risk
Reisand Kallen	3	1	3	7	Low risk
wichman	1	0	2	3	High risk
Bode´n	4	2	3	9	Low risk
Sadowski	4	1	2	8	Low risk
Bellet	4	1	2	8	Low risk
Vigod	4	2	3	9	Low risk
Petersen	4	2	2	8	Low risk
Friedman	3	0	3	6	Low risk
Panchaud	4	2	2	8	Low risk
Frayne	3	1	3	7	Low risk
Park	3	2	4	9	Low risk
Ellfolk1	4	1	3	8	Low Risk
Heinonen	4	2	3	9	Low risk
Kananen	4	2	1	7	Low risk
Kulkarni2023	3	1	1	5	Moderate Risk
Kulkarni2024	3	1	1	5	Moderate Risk

## Discussion

This meta-analysis provides compelling evidence that exposure to SGAs during pregnancy is associated with a moderate but statistically significant increase in GDM risk. Our findings suggest that consumption of SGAs is associated with about 60% higher risk of GDM development (the pooled RR for SGAs was 1.59, whereas FGAs were associated, but not statistically significant, with about 30% higher risk of GDM development (RR = 1.31). Nevertheless, limited number of included studies make us to be cautious when interpreting the effect of various antipsychotic generations on GDM. The highest risk was observed among women with continuous exposure throughout pregnancy, supporting the hypothesis of a dose–time relationship. These findings were consistent across multiple healthcare systems. Compared to the previous meta‐analysis in 2019 by Kucukgoncu et al. (6), which pooled ten studies and found an adjusted RR of 1.30 for any antipsychotic exposure compared to healthy controls, our inclusion of more recent and larger registry‐based studies has helped clarify the differential effects of FGAs vs SGAs. Based on our findings, SGAs show a consistently elevated risk of GDM, whereas FGAs do not. Thus, in women already at high risk of gestational diabetes, FGAs may represent a safer option from a metabolic standpoint, pending consideration of psychiatric need and side‐effect profiles.

The biological plausibility of these findings is well supported. SGAs such as olanzapine and clozapine are known to cause metabolic disturbances via antagonism of histamine H1, serotonin 5-HT2C, and muscarinic M3 receptors, leading to increased appetite, weight gain, and insulin resistance (25). Pregnancy itself is characterized by progressive insulin resistance due to placental hormones; concomitant exposure to SGAs may exacerbate this physiological state, precipitating GDM (26). Park et al. (18) and Heinonen et al. (20) identified a clear gradient of risk, with the highest GDM rates among olanzapine and clozapine exposed pregnancies. Collectively, these findings highlight the clinical significance of cumulative exposure, particularly during the second and third trimesters.

Our results are consistent with large-scale registry data. Ellfolk et al. (19) demonstrated a 43% increased risk of GDM and a higher likelihood of delivering large-for-gestational-age infants among SGA-exposed women. Heinonen et al. (20) refined these findings by stratifying by drug class and trimester, confirming that olanzapine and clozapine carried the greatest risk. In contrast, aripiprazole, which has a more favorable metabolic profile, was not associated with increased GDM risk in Park et al. (18), suggesting that risk is drug-specific rather than uniform across the SGA classes.

Several studies have investigated the impact of treatment continuation versus discontinuation during pregnancy. Wang et al. (27) analyzed cohorts from the UK and Hong Kong and found no significant difference in GDM risk between women who continued antipsychotics during pregnancy and those who discontinued. Lin et al. (28) similarly reported no significant increase in risk with early-pregnancy exposure. These findings suggest that pre-existing maternal risk factors, such as elevated pre-pregnancy BMI, may mediate much of the observed association. Freeman et al. (29) corroborated this by demonstrating that women prescribed SGAs tend to enter pregnancy with higher BMI and greater obesity prevalence, which likely contributes to elevated baseline risk.

This synthesis draws from diverse geographic and clinical contexts, including Sweden, Finland, Canada, the USA, Australia, and Taiwan, thereby enhancing external validity. The inclusion of both registry-based and prospective cohorts minimizes bias, while subgroup and leave-one-out sensitivity analyses confirmed that no single study disproportionately influenced results. Clinically, these findings highlight the importance of targeted glucose screening for pregnant women receiving SGAs and support individualized treatment decisions that balance psychiatric stability against metabolic risk, and also conversion of SGAs to FGAs in high-risk mothers, if possible.

## Limitations

A significant degree of heterogeneity was observed (I2 up to 85%), reflecting differences in study design, patient populations, and exposure definitions. Although most studies adjusted for major confounders, residual confounding from unmeasured variables (e.g., diet, physical activity, illness severity) cannot be excluded. In addition, it is important to note that most studies included did not report drug-specific risks, so we cannot estimate drug-specific risks. Moreover, comorbidities and other confounding factors could also influence GDM risk, and future studies should examine these limitations further. Another limitation of this work is the heterogeneity in some subgroups, which has not been found via meta-regression. Therefore, there is still a need for future primary studies with harmonized designs and standardized exposure measures to clarify sources of heterogeneity and then a dose-response meta-analysis. It is noteworthy to say that there was an inability to evaluate drug-specific risks for gestational diabetes. Although we categorized antipsychotics into first- and second-generation groups, the number of studies providing data for individual drugs (such as olanzapine or quetiapine) was insufficient for meta-analysis. Consequently, our findings should be interpreted as reflecting class-level associations rather than individual drug effects. This limitation restricts the precision of risk estimation for clinical decision-making. Although the results of this study could be a guide for clinicians to choose appropriate medications with last adverse effects, future well-designed studies stratified by specific antipsychotic agents are warranted to better guide clinical practice.

## Clinical implications

Given the increasing global use of antipsychotics, particularly SGAs, among women of reproductive age (24, 30), even modest relative increases in GDM risk translate into significant public health implications. For women receiving SGAs, particularly metabolically high-risk SGAs such as olanzapine and clozapine, early glucose tolerance testing, repeated screening later in pregnancy, and multidisciplinary care are recommended. For patients who are psychiatrically stable on metabolically safer agents (e.g., aripiprazole), continuation during pregnancy may be a reasonable option, with standard GDM monitoring protocols.

## Future research directions

Future research should prioritize prospective studies with precise measurement of pre-pregnancy BMI, gestational weight gain, and antipsychotic dose–response relationships. Advanced methodologies, such as propensity score matching and sibling-comparison designs, may help disentangle medication effects from the influence of underlying psychiatric illness. Biomarker-based studies evaluating insulin sensitivity and placental hormone profiles could provide mechanistic insights. Long-term follow-up of offspring is also warranted to assess intergenerational metabolic effects and guide comprehensive risk–benefit decision-making. Residual confounding remains a major concern in interpreting our findings. Although most included studies adjusted for potential confounders such as maternal age, BMI, preexisting diabetes, parity, smoking status, and psychiatric diagnosis or illness severity, several important variables were often not controlled for. These include family history of diabetes, gestational weight gain, diet, physical activity, and concomitant metabolic medication use. The absence of adjustment for these factors in some of included studies may lead to residual confounding, potentially biasing the estimated association between antipsychotic exposure and gestational diabetes risk. For example, higher baseline metabolic vulnerability or lifestyle factors associated with psychiatric conditions could independently contribute to increased risk, irrespective of drug exposure. Therefore, future studies should address these covariates and are encouraged to use several models to adjust for more viables. This may help us to find the impact of each covariate on GDM. The primary studies should also report unadjusted effect sizes as well.

## Conclusion

Exposure to SGAs during pregnancy, particularly olanzapine and clozapine, is associated with a clinically meaningful increase in GDM risk. This pattern was not seen in FGAs, although a limited number of studies assessing the impact of FGAs on GDM, makes us cautious about our interpretation. Clinicians managing pregnant women who require antipsychotic therapy should adopt a proactive, individualized approach. Early screening for glucose intolerance and finding patients at higher risk of GDM is recommended. Shared decision-making between psychiatrists, obstetricians, and patients is essential to optimize psychiatric stability while minimizing metabolic risk. When clinically appropriate, agents with lower metabolic liability should be preferred, and patients should receive counseling on nutrition, physical activity, and blood glucose monitoring. Integrating these steps into prenatal care can help balance mental health needs with maternal metabolic safety. The risk is amplified with prolonged or multi-trimester exposure and may be partially attributable to pre-existing metabolic vulnerability. These findings highlight the importance of personalized treatment planning, proactive metabolic monitoring, and coordinated perinatal care to optimize maternal and fetal outcomes while preserving psychiatric stability.

## Data Availability

The original contributions presented in the study are included in the article/supplementary material. Further inquiries can be directed to the corresponding author.

## References

[1] Li J Yan J Ma L Huang Y Zhu M Jiang W . Effect of gestational diabetes mellitus on pregnancy outcomes among younger and older women and its additive interaction with advanced maternal age. Front Endocrinology. (2023) 14:1158969. doi: 10.3389/fendo.2023.1158969, PMID: 37234802 PMC10206299

[2] Guo Z Ji W Yan M Zou X Chen T Bai F . Global, regional, and national burden of diabetes in women of childbearing age, 1990-2021: A systematic analysis from the Global Burden of Disease Study 2021. Front Global Women’s Health. (2025) 6:1528661. doi: 10.3389/fgwh.2025.1528661, PMID: 40735640 PMC12303903

[3] Ornoy A Weinstein-Fudim L Ergaz Z . Antidepressants, antipsychotics, and mood stabilizers in pregnancy: what do we know and how should we treat pregnant women with depression. Birth defects Res. (2017) 109:933–56. doi: 10.1002/bdr2.1079, PMID: 28714604

[4] Bodén R Lundgren M Brandt L Reutfors J Kieler H . Antipsychotics during pregnancy: relation to fetal and maternal metabolic effects. Arch Gen Psychiatry. (2012) 69:715–21. doi: 10.1001/archgenpsychiatry.2011.1870, PMID: 22752236

[5] Wang Z Wong ICK Man KKC Alfageh BH Mongkhon P Brauer R . The use of antipsychotic agents during pregnancy and the risk of gestational diabetes mellitus: a systematic review and meta-analysis. psychol Med. (2021) 51:1028–37. doi: 10.1017/S003329171900401X, PMID: 31969198

[6] Kucukgoncu S Guloksuz S Celik K Bahtiyar MO Luykx JJ Rutten BP . Antipsychotic exposure in pregnancy and the risk of gestational diabetes: a systematic review and meta-analysis. Schizophr Bulletin. (2020) 46:311–8., PMID: 31167033 10.1093/schbul/sbz058 PMC7442324

[7] Page MJ McKenzie JE Bossuyt PM Boutron I Hoffmann TC Mulrow CD . The PRISMA 2020 statement: an updated guideline for reporting systematic reviews. bmj. (2021) 372. 10.1136/bmj.n71 PMC8005924 33782057

[8] McKenna K Koren G Tetelbaum M Wilton L Shakir S Diav-Citrin O . Pregnancy outcome of women using atypical antipsychotic drugs: a prospective comparative study. J Clin Psychiatry. (2005) 66:444–9. doi: 10.4088/JCP.v66n0406, PMID: 15816786

[9] Reis M Källén B . Maternal use of antipsychotics in early pregnancy and delivery outcome. J Clin psychopharmacology. (2008) 28:279–88., PMID: 18480684 10.1097/JCP.0b013e318172b8d5

[10] Wichman CL . Atypical antipsychotic use in pregnancy: a retrospective review. Arch women’s Ment Health. (2009) 12:53–7. doi: 10.1007/s00737-008-0044-3, PMID: 19137446

[11] Sadowski A Todorow M Brojeni PY Koren G Nulman I . Pregnancy outcomes following maternal exposure to second-generation antipsychotics given with other psychotropic drugs: a cohort study. BMJ Open. (2013) 3:e003062. doi: 10.1136/bmjopen-2013-003062, PMID: 23852139 PMC3710985

[12] Bellet F Beyens MN Bernard N Beghin D Elefant E Vial T . Exposure to aripiprazole during embryogenesis: a prospective multicenter cohort study. Pharmacoepidemiology Drug Safety. (2015) 24:368–80., PMID: 25683615 10.1002/pds.3749

[13] Vigod SN Gomes T Wilton AS Taylor VH Ray JG . Antipsychotic drug use in pregnancy: high dimensional, propensity matched, population based cohort study. bmj. (2015) 350. doi: 10.1136/bmj.h2298, PMID: 25972273 PMC4430156

[14] Petersen I Sammon CJ McCrea RL Osborn DP Evans SJ Cowen PJ . Risks associated with antipsychotic treatment in pregnancy: comparative cohort studies based on electronic health records. Schizophr Res. (2016) 176:349–56. doi: 10.1016/j.schres.2016.07.023, PMID: 27484686

[15] Hatters Friedman S Moller-Olsen C Prakash C North A . Atypical antipsychotic use and outcomes in an urban maternal mental health service. Int J Psychiatry Med. (2016) 51:521–33. doi: 10.1177/0091217417696739, PMID: 28629296

[16] Panchaud A Hernandez-Diaz S Freeman MP Viguera AC MacDonald SC Sosinsky AZ . Use of atypical antipsychotics in pregnancy and maternal gestational diabetes. J Psychiatr Res. (2017) 95:84–90. doi: 10.1016/j.jpsychires.2017.07.025, PMID: 28810177

[17] Frayne J Nguyen T Bennett K Allen S Hauck Y Liira H . The effects of gestational use of antidepressants and antipsychotics on neonatal outcomes for women with severe mental illness. Aust New Z J Obstetrics Gynaecology. (2017) 57:526–32. 10.1111/ajo.12621 28386942

[18] Park Y Hernandez-Diaz S Bateman BT Cohen JM Desai RJ Patorno E . Continuation of atypical antipsychotic medication during early pregnancy and the risk of gestational diabetes. Am J Psychiatry. (2018) 175:564–74. doi: 10.1176/appi.ajp.2018.17040393, PMID: 29730938 PMC5988929

[19] Ellfolk M Leinonen MK Gissler M Lahesmaa-Korpinen A-M Saastamoinen L Nurminen M-L . Second-generation antipsychotics and pregnancy complications. Eur J Clin Pharmacol. (2020) 76:107–15. doi: 10.1007/s00228-019-02769-z, PMID: 31680189

[20] Heinonen E Forsberg L Nörby U Wide K Källén K . Antipsychotic use during pregnancy and risk for gestational diabetes: a national register-based cohort study in Sweden. CNS Drugs. (2022) 36:529–39. doi: 10.1007/s40263-022-00908-2, PMID: 35220525 PMC9095513

[21] Kananen A Bernhardsen GP Lehto SM Huuskonen P Kokki H Keski-Nisula L . Quetiapine and other antipsychotic medications during pregnancy: a 15-year follow-up of a university hospital birth register. Nordic J Psychiatry. (2023) 77:651–60. doi: 10.1080/08039488.2023.2205852, PMID: 37149788

[22] Kulkarni J Gurvich C Gilbert H Worsley R Li Q Karimi L . The use of first and second-generation antipsychotic drugs and the potential to develop gestational diabetes mellitus among perinatal patients with psychosis. Schizophr Res. (2023) 254:22–6. doi: 10.1016/j.schres.2023.01.030, PMID: 36758325

[23] Kulkarni J De Chellis A Gilbert H Gavrilidis E Mu E Karimi L . Clozapine safety in pregnancy: A clinical study. Schizophr Bull. (2024), sbae132. doi: 10.1093/schbul/sbae132, PMID: 39031964 PMC12996916

[24] Park Y Huybrechts KF Cohen JM Bateman BT Desai RJ Patorno E . Antipsychotic medication use among publicly insured pregnant women in the United States. Psychiatr Serv. (2017) 68:1112–9., PMID: 28617210 10.1176/appi.ps.201600408 PMC5665733

[25] Mukherjee S Skrede S Milbank E Andriantsitohaina R López M Fernø J . Understanding the effects of antipsychotics on appetite control. Front Nutr. (2022) 8:815456. doi: 10.3389/fnut.2021.815456, PMID: 35047549 PMC8762106

[26] Mittal R Prasad K Lemos JR Arevalo G Hirani K . Unveiling gestational diabetes: an overview of pathophysiology and management. Int J Mol Sci. (2025) 26:2320. doi: 10.3390/ijms26052320, PMID: 40076938 PMC11900321

[27] Wang Z Man KKC Ma T Howard LM Wei L Wong ICK . Association between antipsychotic use in pregnancy and the risk of gestational diabetes: Population-based cohort studies from the United Kingdom and Hong Kong and an updated meta-analysis. Schizophr Res. (2021) 229:55–62. doi: 10.1016/j.schres.2020.11.021, PMID: 33243714

[28] Lin HY Lin FJ Katz AJ Wang IT Wu CH . Antipsychotic use in early pregnancy and the risk of maternal and neonatal complications. Mayo Clin Proc. (2022) 97:2086–96. doi: 10.1016/j.mayocp.2022.04.006, PMID: 36210203

[29] Freeman MP Sosinsky AZ Goez-Mogollon L Savella GM Moustafa D Viguera AC . Gestational weight gain and pre-pregnancy body mass index associated with second-generation antipsychotic drug use during pregnancy. Psychosomatics. (2018) 59:125–34. doi: 10.1016/j.psym.2017.09.002, PMID: 29078988

[30] Reutfors J Cesta CE Cohen JM Bateman BT Brauer R Einarsdóttir K . Antipsychotic drug use in pregnancy: A multinational study from ten countries. Schizophr Res. (2020) 220:106–15. doi: 10.1016/j.schres.2020.03.048, PMID: 32295750 PMC7306443

